# Circulating M-MDSC Levels as an Assessment Marker for Post-Treatment Tumor Progression in Recurrent HNC Patients Following Radiation Therapy: A Case Series

**DOI:** 10.3390/jcm13175130

**Published:** 2024-08-29

**Authors:** Chun-Hsiang Chang, Fang-Hsin Chen, Ling-Wei Wang, Chi-Shiun Chiang

**Affiliations:** 1 Department of Biomedical Engineering and Environment Sciences, National Tsing Hua University, Hsinchu 300044, Taiwan; doo1002000@yahoo.com.tw; 2 Institute of Nuclear Engineering and Science, National Tsing Hua University, Hsinchu 300044, Taiwan; fanghsin@mx.nthu.edu.tw; 3 Department of Heavy Ion and Radiation Oncology, Taipei Veterans General Hospital, Taipei 112304, Taiwan; 4 School of Medicine, National Yang-Ming Chiao Tung University, Taipei 30010, Taiwan; 5 Boron Neutron Capture Therapy Center, National Tsing Hua University, Hsinchu 300044, Taiwan

**Keywords:** recurrent HNC, M-MDSCs, BNCT, IG-IMRT

## Abstract

**Background**: In advanced head and neck cancer (HNC) patients, 50–60% experience loco-regional relapse and distant metastasis. Boron neutron capture therapy (BNCT) has shown remarkable therapeutic response in recurrent HNC, but there is still a 70% chance of local recurrence. This study aimed to identify a suitable liquid biomarker to assess patient response following BNCT. Myeloid-derived suppressor cells (MDSCs) are immune-suppressive cells that inhibit cytotoxic T cells. Circulating MDSC levels have been linked to the clinical stage and prognosis in HNSCC. **Methods**: Five patients with recurrent head and neck cancer underwent a treatment regimen that commenced with BNCT, followed by fractionated image-guided intensity-modulated radiotherapy (IG-IMRT). Liquid biopsy analysis via flow cytometry and tumor volume analysis by clinical imaging were conducted at three stages: before BNCT, before the first fraction of IG-IMRT, and one month after the last fraction of IG-IMRT. **Results**: Compared to other MDSC subtypes, monocytic MDSCs (M-MDSCs) exhibited a notable correlation with tumor volume. This strong correlation was observed at all testing time points except one month after BNCT treatment. **Conclusions**: This case series highlights a strong link between tumor size and circulating M-MDSC levels before BNCT and one month after the last IG-IMRT treatment in recurrent head and neck cancer patients. These results suggest that the level of circulating M-MDSCs could be a marker for monitoring tumor progression in recurrent HNC patients following radiation therapy, including BNCT.

## 1. Introduction

Head and neck cancer (HNC) is the eighth most common cancer worldwide. In this cancer, around 90% of cases are squamous cell carcinoma. Alcohol consumption and tobacco smoking are the two most significant risk factors for the development of HNC. Additionally, infections with oncogenic viruses, such as human papillomavirus (HPV) and Epstein–Barr virus (EBV), have been linked to the development and progression of these neoplasms [1,2]. The treatment depends on the location, size, status, and stage of the tumor and lymph node and specializes in variable combinations of surgery, chemotherapy [3], radiotherapy, targeted therapy, and immune therapy. Despite the advances in diagnosis and treatment over the past three decades, up to 50–60% of patients with locally advanced disease experience loco-regional relapse and distant metastasis. Image-guided intensity-modulated radiotherapy (IG-IMRT) was used for salvage therapy with a 2-year local control rate of around 50% [4]. Recurrence in HNC often proves to be resistant to standard treatments like radiotherapy and chemotherapy because the normal tissue surrounding the tumor has previously received a certain degree of radiation. This imposes limitations on the radiation dose it can tolerate for the recurrent tumor and prompts a need for novel therapeutic approaches.

Boron neutron capture therapy (BNCT) is a form of high-linear energy transfer (LET) radiation known for its limited range, enabling it to target tumor cells primarily due to their preferential boron accumulation while sparing the adjacent normal tissue [5]. BNCT has been used for treating recurrent HNC and malignant brain tumors and resulted in a notable reduction in tumor size ranging from 46% to 100% [6,7]. Previous clinical trials in Taiwan using THOR-based BNCT for recurrent HNC patients achieved a high response rate of approximately 70% and extended median survival to 19.9 months [8,9]. However, for some large recurrent tumors, it was difficult to obtain durable tumor control by BNCT alone [10]. Fractionated photon radiotherapy with an intensity modulation technique can also obtain conformal dose distribution around the tumor and a lower dose to the surrounding normal tissue. A combination of BNCT with photon beams and an IG-IMRT approach may further improve local control and limit toxicity to an acceptable level for recurrent HNC. To procure better tumor control, we started another clinical trial combining BNCT and IG-IMRT in 2014 [11].

Myeloid-derived suppressor cells (MDSCs) are a heterogeneous group of immune-suppressive cells originating from the myeloid lineage. MDSCs in humans have the phenotypes of CD33^+^, CD11b^+^, and HLA-DR^−/low^ [12]. They encompass three primary subtypes, PMN-MDSCs (polymorphonuclear MDSCs), M-MDSCs (monocytic MDSCs), and e-MDSCs (early MDSCs), distinguishable by the presence of CD14 or CD15 [13,14]. MDSCs are involved in multiple aspects of immune regulation in conditions such as chronic inflammation, infection, autoimmune diseases, trauma, graft-versus-host disease, and particularly cancer [15]. MDSCs are known for their ability to dampen immune responses, creating an immunosuppressive environment within the tumor microenvironment that facilitates cancer progression [16,17]. Reports have shown that the MDSC levels in patients’ peripheral blood are significantly associated with advanced clinical stages and a poorer prognosis of HNC [18,19]. However, variability in MDSC levels following radiation therapy has not been reported in clinics. We hypothesized that the level of circulating MDSCs could be a marker for monitoring tumor progression in recurrent HNC patients following radiation therapy.

This study aimed to explore the relationship between blood M-MDSC levels and tumor volumes in our clinical trial’s participants.

## 2. Materials and Methods

### 2.1. Patients

From November 2019 to January 2022, five patients with previously irradiated recurrent head and neck tumors received BNCT treatment at the Tsing Hua Open Reactor (THOR) facility, followed by IG-IMRT for one month after one month of BNCT at Taipei Veterans General Hospital for salvage treatment. All participants were in life-threatening conditions and met the Emergency and Compassionate Use criteria, as approved by the institutional review board of Taipei Veterans General Hospital (Approval number: 2012-06-016A). Written informed consent was obtained from all patients. The tumor size and blood samples were assessed at three time points: before BNCT treatment, one month after BNCT, and one month after the therapeutic process.

### 2.2. BNCT and IG-IMRT Administration

An intravenous infusion of 500 mg/kg L-BPA–F (Hammercap AB, Sweden, and Taiwan Biopharm Company, Taoyuan, Taiwan) was administered in two phases. The first phase involved an infusion rate of 200 mg/kg/h for 2 h before neutron irradiation, followed by a second phase at 100 mg/kg/h during irradiation, which was halted when the beam was turned off. The epithermal neutron source for BNCT was provided by the Tsing-Hua Open-Pool Reactor (THOR) at National Tsing-Hua University. The blood boron concentration during BNCT was estimated using measurements immediately before and after irradiation by inductively coupled plasma optical emission spectrometry (ICP-OES, Avio 200, PerkinElmer, Shelton, CT, USA). Daily fractionated IG-IMRT was performed using a TomoTherapy Hi-ART system (Accuray, Sunnyvale, CA, USA) and was initiated one month after BNCT. The tumor dose was maximized to the highest achievable level within the acceptable range for critical organs.

### 2.3. Tumor Stage

The tumor stage was determined according to the AJCC 8th edition guidelines.

### 2.4. Tumor Size

PET/CT was employed to identify and confirm tumor lesions in patients, utilizing 18F-FDG positron emission tomography (PET) to enhance the visibility of tumor lesions. The tumor volumes were obtained during contouring with Eclipse Ver. 13.6 (Varian, Palo Alto, CA, USA) before BNCT and IG-IMRT. The third measurement was performed one month after IG-IMRT for the initial response using the same software. For the evaluation of the true tumor response, a PET scan was conducted three months after IG-IMRT to avoid false positivity.

### 2.5. Tumor Response

All patients were followed up with a PET/CT or CT examination to assess tumor progression. One month after the therapeutic process, a PET/CT or CT examination was performed to determine the volume changes and define the tumor response. The assessment of the tumor response followed the guidelines of RECIST (Response Evaluation Criteria in Solid Tumors) version 1.1. The evaluation of target lesions included CR (complete response): disappearance of all target lesions, all nodal lesions have short axis less than 10 mm; PR (partial response): 30% or more decrease in the sum of diameters from baseline sum diameters; PD (progression of disease): 20% or more increase in the smallest sum of diameters as reference with an absolute increase of more than or equal to 5 mm; SD (stable disease): does not meet the above criteria.

### 2.6. Flow Cytometry

Blood samples were collected from patients and treated with red blood cell lysis buffer (00-4300-54, eBioscience, San Diego, CA, USA) for 5 min at room temperature. The remaining blood cells were blocked with Human TruStain FcXTM (422302, BioLegend, San Diego, CA, USA) and labeled with the following antibodies: anti-human Lineage (CD3, CD19, CD20, and CD56)-APC (363601, BioLegend), HLA-DR-FITC (307604, BioLegend), CD11b-PerCp Cy5.5 (301328, BioLegend), CD14-PE (325606, BioLegend), and CD15-PE/Cy7 (560827, BD Biosciences, San Jose, CA, USA). The stained cells were analyzed using FACSCanto flow cytometry. MDSCs were identified as Lin-HLA-DR-CD11b+ cells, with CD14 and CD15 used to classify PMN-MDSCs (CD14−CD15+), M-MDSCs (CD14+CD15+), and e-MDSCs (CD14−CD15−). Data acquisition was performed with BD FACSDiveTM software, and analysis was conducted using FACSDivaTM ver 9.0 and FlowJo version 10.6.2.

### 2.7. Statical Analysis

GraphPad Prism version 8.3.0 (GraphPad Software) generated all statistical analyses and graphs. P values less than 0.05 were considered statistically significant (* *p* < 0.05, ** *p* < 0.01, *** *p* < 0.001, **** *p* < 0.0001).

## 3. Results

**Figure 1:**
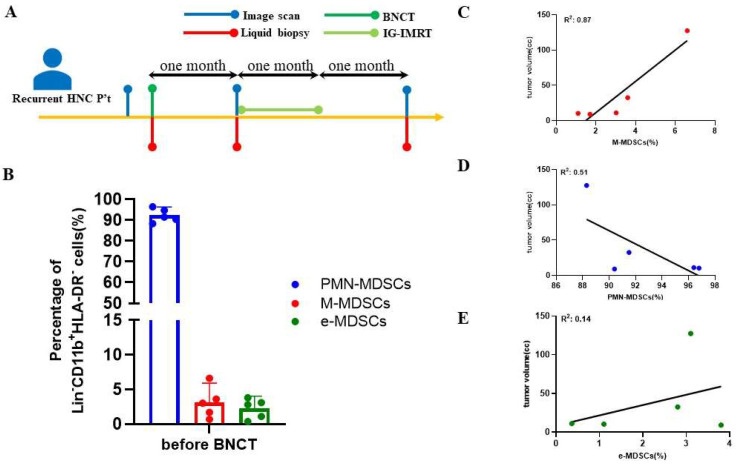
Association between MDSC population and tumor size in patients with recurrent HNC before treatment. (**A**) The schedule of the therapeutic process. (**B**) MDSC population in patients with recurrent HNC. Correlations between tumor volume and (**C**) M-MDSCs, (**D**) PMM-MDSCs, and (**E**) e-MDSCs. Correlation analysis was fitted with a simple linear regression.

**Figure 2:**
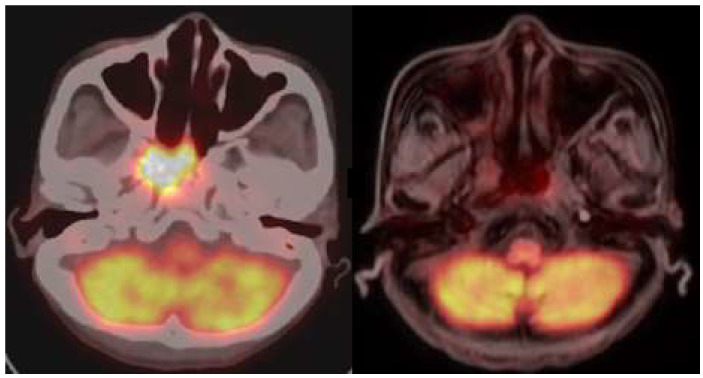
Imaging results for patient 5. (**Left**) The positron emission tomography (PET)/computed tomography (CT) image of patient 5 was taken before BNCT treatment. (**Right**) The PET/magnetic resonance imaging (MRI) scan displayed tumor recurrence in patient 5 following the complete treatment for three months.

The participants in this study comprised five patients with recurrent HNC. Their demographic information is presented in Table 1 and Table 2. The adverse effects induced by the treatment are presented in Table 3. Patients underwent BNCT followed by fractionated IG-IMRT treatment one month after BNCT. PET/CT scans were utilized to confirm tumor size and treatment response before BNCT, IG-IMRT, and after the completion of the IG-IMRT procedure. The levels of circulating MDSCs were measured by flow cytometry, synchronized with the timing of PET/CT scans. The tumor volume was calculated through 3D image reconstruction from CT images. Before the BNCT treatment, the average percentage levels of PMN-MDSCs, M-MDSCs, and e-MDSCs among MDSCs were 91.23 ± 2.67%, 3.15 ± 2.60%, and 2.70 ± 1.15%, respectively (Figure 1A). To examine the relationship between tumor size and MDSC levels, a correlation analysis was conducted using Spearman’s rank correlation coefficient, and simple linear regression was applied for curve fitting. A significantly positive correlation with tumor volume in patients with recurrent HNC before BNTC treatment was found with the level of circulating M-MDSCs (Figure 1B) but not with the levels of PMN-MDSCs and e-MDSCs (Figure 1C,D). One representative case (patient 1) was demonstrated. A 41-year-old female with recurrent nasopharyngeal cancer underwent single-fraction BNCT with a 16.4 Gy(w) dose on 13 December 2019, followed by 50 Gy fractionated IG-IMRT (25 fractions) from 13 January 2020 to 19 February 2020. Liquid biopsy and PET scans were conducted before BNCT, pre-IG-IMRT, and three months post-IG-IMRT. Pre-IG-IMRT imaging showed a slight tumor size reduction, while post-IG-IMRT imaging confirmed a complete response (CR). Patient 5 also demonstrated the same remarkable therapeutic effect (Figure 2). However, a one-year follow-up of patient 1 showed a sudden increase in M-MDSC levels, and subsequent CT scans revealed tumor recurrence. This patient received subsequent targeted therapy and remains alive as of the writing of this manuscript.

**Figure 3:**
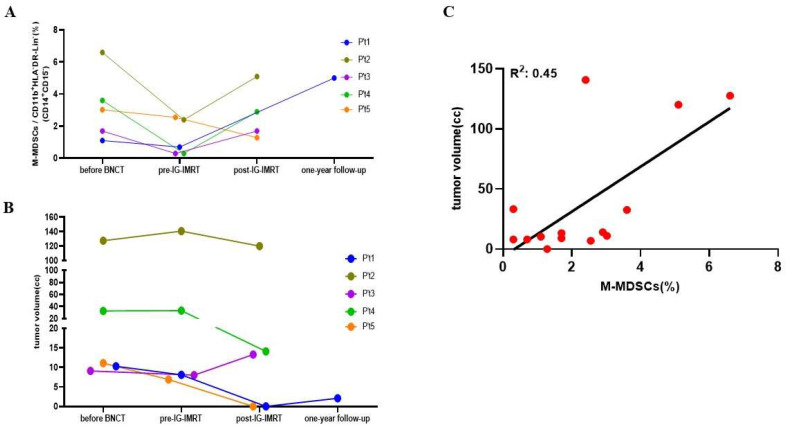
The change in circulating M-MDSCs and tumor volume at different time points. (**A**) The fluctuations in circulating M-MDSCs throughout treatment. (**B**) The variations in tumor volume throughout treatment. (**C**) The correlation between circulating M-MDSCs and tumors. The correlation between circulating M-MDSCs and tumor size in the entire process.

Figure 1 shows a good correlation between the levels of circulating M-MDSCs and the tumor burden before BNCT; both elements were evaluated at two other specific time points: one month after BNCT (pre-IG-IMRT) and one month after the completion of IG-IMRT (post-IG-IMRT). The levels of circulating M-MDSCs in all patients at the pre-IG-IMRT time point decreased in comparison to their previous values (Figure 3A). However, the tumor size at pre-IG-IG-IMRT did not exhibit a significant change among all patients (Figure 3B). There is no correlation between the tumor size and M-MDSC levels at this stage (Supplementary Figure S1). Among these patients, two patients experienced disease progression (patients 2 and 4), two patients showed a reduction in tumor size (patients 1 and 5), and one patient’s tumor size was unchanged (patient 3).

When the level of circulating M-MDSCs one month after the completion of the last IG-IMRT was analyzed (Figure 3A), the M-MDSC levels in three patients increased (patients 2, 3, and 4), and in one patient, the level decreased further (patient 5). Patient 1 attained a complete response at the pre-IG-IMRT time, and we failed to detect the level of MDSCs. Although patient 1 had a CR one month after the therapeutic process, the M-MDSCs increased in the 1-year follow-up period. A recurrent tumor was then noted at the treatment site. When the correlation between the level of M-MDSCs and the tumor volume of these four patients at this time point was analyzed, a linear correlation was observed with R^2^ = 0.89 (Supplement Figure S1) despite the lack of statistical significance due to the low patient number. However, these data suggest a correlation between the tumor volume and M-MDSC levels in recurrent HNC patients. The results revealed a good correlation between the M-MDSC level and tumor size with the goodness of fit having an R^2^ value of 0.85, with the data from patients before BNCT and one month after the last fraction of IG-IMRT. If all data from pre-treatment, pre-IG-IMRT, and post-treatment were analyzed, the goodness of fit decreased to 0.45 (Figure 3C). Nevertheless, these results indicated that M-MDSC levels were significantly associated with tumor progression in patients with recurrent HNC, not only before BNCT but also after IG-IMRT.

## 4. Discussion and Conclusions

In Taiwan, two clinical trials have shown a high response rate of recurrent HNC to BNCT, improving the quality of life [10,11]. The first clinical trial used two fractions of BNCT [10]. The trial demonstrated that two fractions of BNCT were effective and safe for recurrent HNC. However, the recurrence around BNCT re-irradiated sites was frequently reported in the follow-up study. The second trial using IG-IMRT as a salvage therapy following BNCT was designed to achieve a larger field around the recurrent gross tumor volume. It was hypothesized that IG-IMRT could improve local control with acceptable toxicity. This protocol recruited 14 patients and concluded that there was a high response rate and low incidence of grade 4 toxicity [11]. Among the 14 patients recruited for the BNCT + IG-IMRT trial, only 7 were registered for blood MDSCs analysis in this study. It is therefore necessary to remember that the response rate reported in this study did not faithfully represent the response rate of the BNCT plus IG-IMRT trial for recurrent HNC.

Despite the notable therapeutic response of BNCT in recurrent HNC patients, many patients still suffer from recurrence or metastasis. The primary challenge lies in the absence of precise methodologies for detecting recurrence earlier and monitoring disease progression in these patients. It has been shown that the levels of circulating MDSCs in HNC patients were higher than in healthy donors and positively related to advanced tumor progression [19]. To verify the therapeutic effect of BNCT and eliminate the impact of IG-IMRT, we measured the tumor size one month after BNCT treatment [11], rather than the conventional three-month period. The presented cases show a strong correlation between tumor size and M-MDSCs in recurrent HNC patients, both pre- and post-treatment. These results indicated that monitoring M-MDSC proportions in the blood of recurrent HNC patients could be a valuable tool for assessing tumor response following BNCT. Although imaging is the current standard protocol to evaluate the therapeutic effect, given the correlation between the levels of M-MDSCs and tumor size found in this study, and the significant increase in M-MDSCs observed during tumor recurrence following the complete therapeutic process, blood M-MDSC levels can also be a biomarker for evaluating the response and predicting recurrence after therapy. If more clinical data confirm this finding, monitoring blood M-MDSC levels will be much cheaper and easier than using imaging techniques. If it can predict recurrence earlier, it gives clinicians more time and options to prevent or delay recurrence. Even during the early evaluation (one month after BNCT) in this pilot study, we saw a progression of disease with obvious tumor size rebound (patient 3 in Figure 3B), and the patient died 10 days later. For better correlation with tumor response, further M-MDSC measurements should be repeated 3 months after IG-IMRT.

Despite a strong correlation between tumor size and M-MDSCs, serial cases show a decrease in M-MDSCs one month after BNCT without a significant association in tumor size or patient response. However, the M-MDSC level reduction was not further observed one month after IG-IMRT follow-up therapy in this case series report. Although circulating M-MDSCs cannot predict the therapeutic response in advance, observing Case 1 consistently showed a complete response post-treatment for almost a year, followed by recurrence after a year. Simultaneously, the M-MDSC population reached 5%, surpassing the average M-MDSC levels among all patients. A good correlation was observed when the data one month after BNCT were excluded from the correlation analysis. This finding indicates that a transient reduction in M-MDSC levels may be a unique response to BNCT. Although this transient reduction and subsequent recovery in MDSC levels were also observed in HNC patients following chemotherapy, their association with tumor volume was not reported [20]. In the mouse subcutaneous SCC7 tumor model, administering hypofractionated radiation or PD-L1 antibodies alone did not reduce the proportion of M-MDSCs in the blood but rather increased it [21]. This BNCT-induced unique M-MDSC response is consistent with our previous study in a murine 4-NQO-induced oral cancer tumor model [22].

Previous studies on oral squamous cell carcinoma (OSCC) have shown elevated levels of PMN-MDSCs, which inhibit T-cell function [23]. This study indicates that M-MDSC levels correlate more strongly with recurrent head and neck cancer (HNC) tumor volume than PMN-MDSCs. Additionally, our previous study in a 4-NQO-induced tumor model demonstrated that targeting circulating M-MDSCs can enhance the therapeutic effectiveness of BNCT associated with the increase in infiltrating CD8+ T cells [22]. These results suggest the role of M-MDSCs in promoting tumor progression and the potential for utilizing this combination therapy to improve tumor control rates in patients with recurrent HNC.

The limitation of this clinical case is the role of M-MDSCs. We did not explore the variability in the circulating T-cell population simultaneously. Analyzing the proportion and activity of immune cells in the bloodstream could offer a more comprehensive understanding of BNCT’s effect on M-MDSCs. Since BNCT is not a routine treatment for recurrent HNC despite its excellent therapeutic effects, this case series includes only a small number of patients. The limited sample size makes it challenging to interpret differences among the patients. Recent studies have shown that tumors located in the mucosa [24] and patients receiving a minimum dose of more than 18 Gy(w) [25] achieve better therapeutic efficiency with BNCT. However, this small number of case series indicates that tumors in the Nasopharynx respond better to BNCT than in the oral cavity. These two cases are also the only two EBV-positive cases. However, it is too early to conclude this case series; including more patients and parameters is encouraged to evaluate the association between the response to BNCT and these parameters.

Regarding the dose of this clinical trial, Memorial Sloan Kettering Cancer Center used proton therapy of 60 to 70 Cobalt Gray Equivalent for previously irradiated head neck cancer [26,27]. BNCT spared normal tissue better than proton therapy. Furthermore, in view of the rest period of 28 days between BNCT and IG-IMRT, we may have used similar biological doses compared with their practice. They observed a few cases of grade 5 toxicity, which we did not have in this trial [11].

In summary, this case series revealed a significant correlation between circulating M-MDSC frequency and recurrent tumor volume. This underscores the potential of M-MDSCs as markers for assessing post-treatment tumor progression and as potential therapeutic targets in recurrent HNC patients following radiation therapy. Due to the ease of measuring blood M-MDSC levels, this report suggests more clinical studies to confirm this finding.

## Figures and Tables

**Table 1:** Patient demographics.

	Characteristics	n (%)	Median (Range)
Patient			
	Gender		
	Male	3 (60)	
	Female	2 (40)	
	Age (y)		51 (41, 74)
Tumor			
	Primary site		
	Oral cavity	3 (60)	
	Nasopharynx	2 (40)	
	Volume (cc)		11.1 (9.1, 127.5)
	Recurrent T stage		
	rT3	2 (40)	
	rT4	3 (60)	
	Recurrent N stage		
	rN0	4 (80)	
	rN1	1 (20)	
	BNCT		
Average equivalent tumor dose	(Gy (Eq))		16.9 (13.4, 19.2)
	IG-IMRT		
	Total prescription dose		48 (41.4, 53.0)
	Total fractions		25 (23, 26)
Response			
	CR	2 (40)	
	PR	1 (20)	
	PD	2 (40)	

Tumor response was assessed using Response Evaluation Criteria in Solid Tumors (RECIST) v1.1: complete response (CR), partial response (PR), and progression of disease (PD).

**Table 2:** Individual patient information.

Patient	1	2	3	4	5
Gender	F	M	M	M	F
Age	41	57	50	51	75
Primary tumor	Nasopharynx	Oral cavity	Oral cavity	Oral cavity	Nasopharynx
	T4N3M0	T4aN3bM0	T4aN1M0	T3N0M0	T3N2M0
Recurrent site	Nasopharynx	Right maxilla/temporal	Left maxilla	Right chin	Nasopharynx
	T3N0M0	T4bN0M0	T4N0M0	T3N1M0	T4N0M0
Previous treatment	Induction chemo + CCRT	Surgery + CCRT	CCRT	Surgery + CCRT	CCRT
Irradiation field/dose	Nasopharynx (69.3 Gy/33 fx) Left neck (59.4 Gy/33 fx) and right neck (52.8 Gy/33 fx)	Right upper gingiva(70 Gy/35 fx) Right upper neck (63 Gy/35 fx) and right lower neck (50.4 Gy/28 fx)	Primary left buccal tumor and neck LAPs (70 Gy/35 fx) Peribuccal area and bil upper neck (63 Gy/35 fx)	Primary tongue surgical bed (66 Gy/30 fx) Whole tongue (60 Gy/30 fx) and bilateral neck (54 Gy/35 fx)	Nasopharynx (70 Gy/35 fx) Right upper neck (63Gy/35fx) Right lower neck and left neck (50.4 Gy/28 fx)
PD-L1 or another cytotoxic drug	Chemotherapy with Cisplatin + De Gramont	Chemotherapy with TPFx2 PD-L1 inhibitor with Pembrolizumab	Lenvatinib and Pembrolizumab	Chemotherapy with Erbitux + Cisplatin + 5FU PD-L1 inhibitor with Pembrolizumab Chemotherapy with Taxol + Cisplatin	Nil.
BNCT dosage (mean, Gy(w))	16.4	13.4	16.9	17.5	18.7
IG-IMRT dosage (Gy)	50	48	46.8	53	41.4
Fractions	25	24	26	25	23
Response	CR	PD	PD	PR	CR
EBV (Epstein–Barr Virus)	-	-	-	-	+
HPV (Human papilloma virus)	-	-	-	-	-
Prognosis (From November 2019 to January 2022) with tumor, without tumor, or death	Alive with cancer	Dead with cancer	Dead with cancer	Dead with cancer	Alive with cancer

Tumor response was assessed using Response Evaluation Criteria in Solid Tumors (RECIST) v1.1: complete response (CR), partial response (PR), and stable disease (SD).

**Table 3:** Adverse effects.

Patient Acute	1	2	3	4	5
Mucositis	2	1	1	2	2
Radiation dermatitis	1	1	1	1	1
Alopecia	1	1	1	1	1
Dysphagia	1	1	N/A	N/A	0
Tumor pain	1	0	1	2	0
Hemorrhage	0	1	0	0	0
Infection (soft tissue)	0	3	0	1	0
Edema (H&M)	1	0	0	0	0
Edema (laryngeal)	0	0	0	0	0
Otitis	0	0	0	0	0
Nausea or vomiting	0	0	0	0	0
Trismus	0	0	0	2	0
* Chronic					
Osteoradionecrosis	2	N/A	0	3	2
Soft tissue necrosis	2	N/A	2	2	0
Hemorrhage	0	N/A	0	4	0
Cranial neuropathy	3	N/A	0	0	2
Pain	2	N/A	2	3	2
Skin ulceration	0	N/A	3	3	0
Alopecia	0	N/A	0	0	0
Impaired hearing	2	N/A	0	0	2
Otitis	2	N/A	0	0	2
Xerostomia	0	N/A	0	0	1
Edema (H&M)	0	N/A	0	0	0
Trismus	2	N/A	0	0	0
Infection	2	N/A	0	3	2

Adverse effects that developed beyond 3 months after the last fraction of BNCT. * Adverse effects that developed beyond 3 months after the last fraction of IG-IMRT.

## Data Availability

The original contributions presented in the study are included in the article, further inquiries can be directed to the corresponding author/s.

## Supplementary Materials

The following supporting information can be downloaded at https://www.mdpi.com/article/10.3390/jcm13175130/s1. Figure S1. The correlation between circulating M-MDSCs and tumor size at various time points. Left figure: M-MDSCs exhibited a non-significant correlation with tumor size at pre-IMRT. Right figure: M-MDSCs displayed a notable correlation with tumor size at post-IMRT. Figure S2. Limited association between tumor size and PMN-MDSCs or e-MDSCs. Correlations between tumor volume and PMM-MDSCs before treatment (A), after the complete process for one month (B), throughout the process (C), and before BNCT plus post-IMRT (D). Correlations between tumor volume and e-MDSCs before treatment (E), after the complete process for one month (F), throughout the process (G), and before BNCT plus post-IMRT (H).

## Abbreviations

HNC: head and neck cancer; BNCT: Boron neutron capture therapy; M-MDSCs: monocytic-myeloid-derived suppressor cells; IG-IMRT: Image-guided intensity-modulated radiotherapy; MRI: Magnetic Resonance Imaging; PET: positron emission tomography; CT: computed tomography; LET: linear energy transfer; THOR: Tsing Hua Open Reactor; MDSCs: Myeloid-derived suppressor cells; PMN-MDSCs: polymorphonuclear-MDSCs; e-MDSCs: early-MDSCs.
