# Supplementary material for: Circulating M-MDSC Levels as an Assessment Marker for Post-Treatment Tumor Progression in Recurrent HNC Patients Following Radiation Therapy: A Case Series
Source: J Clin Med. 2024 Aug 29;13(17):5130. doi: 10.3390/jcm13175130 (PMC11396399; doi:10.3390/jcm13175130)
Supplement: Supplementary file 1 [file jcm-13-05130-s001.zip › jcm-3140102-supplementary.pdf]

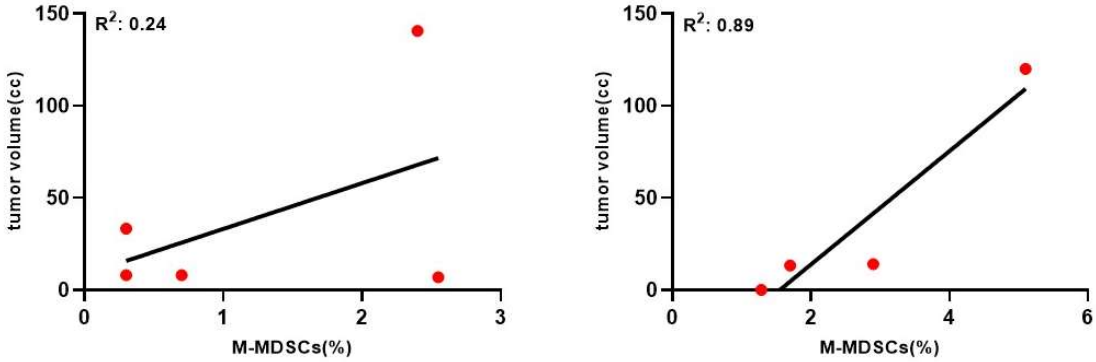

Figure S1. The correlation between circulating M-MDSCs and tumor size at various time points.Left figure: M-MDSCs exhibited a non-significant correlation with tumor size at pre-IMRT. Right figure: M-MDSCs displayed a notable correlation with tumor size at post-IMRT.

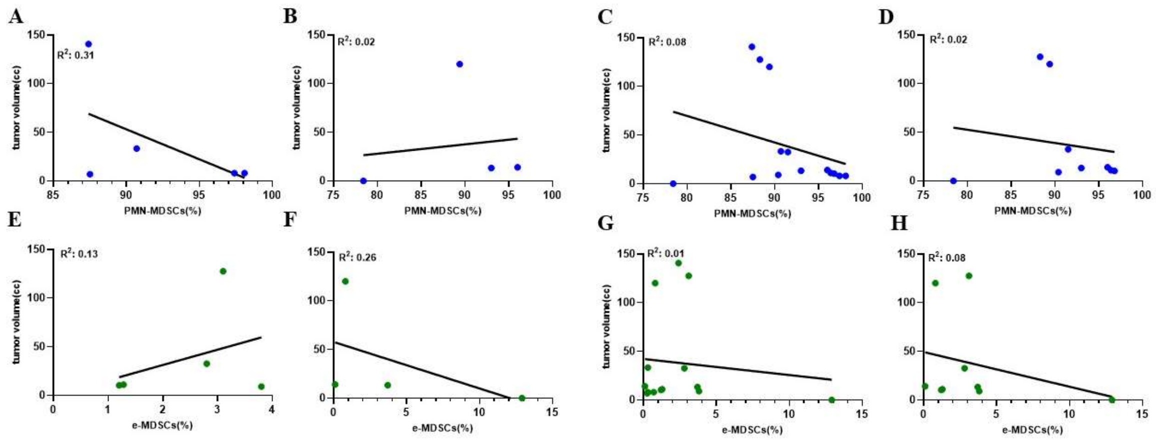

Figure S2. Limited association between tumor size and PMN-MDSCs or e-MDSCs.Correlations between tumor volume and PMM-MDSCs before treatment (A), after the complete process for one month (B), throughout the process (C), and before BNCT plus post-IMRT (D). Correlations between tumor volume and e-MDSCs before treatment (E), after the complete process for one month (F), throughout the process (G), and before BNCT plus post-IMRT (H).
